# Development and Validation of a Short Questionnaire on Dietary and Physical Activity Habits for Patients Submitted to Bariatric Endoscopic Therapies

**DOI:** 10.1007/s11695-021-05754-7

**Published:** 2021-10-19

**Authors:** Gemma Miranda-Peñarroya, Marta Vallejo-Gracia, Ana-Maria Ruiz-León, Fernando Saenger-Ruiz, Ricardo Sorio-Fuentes, Maria Izquierdo-Pulido, Andreu Farran-Codina

**Affiliations:** 1 Clínica Opción Médica S.L, Barcelona, Spain; 2 Departament d’Infermeria Fonamental i Medicoquirúrgica de La Facultat de Medicina, Universitat de Barcelona, Barcelona, Spain; 3 Departament de Nutrició, Ciències de L’Alimentació i Gastronomia, Universitat de Barcelona, Campus de l’Alimentació de Torribera, Santa Coloma de Gramenet, 08921 Barcelona, Spain; 4 Departament de Medicina Interna, Hospital Clínic, Institut d’Investigació Biomèdica August Pi i Sunyer (IDIBAPS), Universitat de Barcelona, Barcelona, Spain; 5 CIBER de Fisiopatología de la Obesidad y la Nutrición (CIBEROBN), Instituto de Salud Carlos III, Madrid, Spain; 6 Institut de Recerca en Nutrició i Seguretat Alimentaria (INSA), Universitat de Barcelona, Barcelona, Spain

**Keywords:** Obesity, Bariatric endoscopic therapies, Short questionnaire, Dietary habits, Physical activity habits

## Abstract

**Purpose:**

Individuals with obesity frequently regain weigh after endoscopic bariatric therapies (EBT) unless they adhere to healthy habits. The objective was to create and validate a short, self-administered questionnaire (EMOVE) to assess healthy dietary and physical activity (PA) habits’ adherence to be used in clinical practice.

**Materials and Methods:**

In this prospective, unicentric study, 463 patients completed the short, Spanish EMOVE questionnaire, to be validated following the Medical Outcome Trust Criteria. Conceptual and measurement model, reliability (internal consistency and test–retest [subgroup of 93 patients]), construct validity, responsiveness, interpretability, and burden were evaluated. Patients enrolled from January 2017 through August 2018 and auto-filled the EMOVE at baseline and at 3, 6, and 12 months.

**Results:**

Patients submitted to intragastric ballon for 6 and 12 months or POSE were 82.7% women with a mean age of 42.7 years, and a mean BMI of 37.1 kg/m^2^. Four factors were extracted with exploratory factor analysis related to intake frequency, portions and proportions, time and place of eating, and physical activity. EMOVE showed adequate internal consistency (*α* = 0.73), very good test–retest (*r* = 0.91, CI: 0.86–0.94; *p* < 0.001), moderate construct validity of dietary (*r* = 0.24, CI: 0.11–0.37, *p* < 0.001), and PA habits (*r* = 0.44, CI 0.30–0.58; *p* < 0.001). Stable responsiveness, with correlations from 0.29 to 0.39 (*p* < 0.001) between the EMOVE scores and the % of total weight loss at 3, 6, and 12 months. Participants categorized as having good or excellent habits (score ≥ 30 points) lost significantly more weight (*p* < 0.05). Finally, the administration burden was 2.96 min.

**Conclusion:**

The EMOVE is a useful tool in Spanish language to easily assess the level of adherence to healthy dietary and PA habits to be used routinely in clinical practice.

**Graphical abstract:**

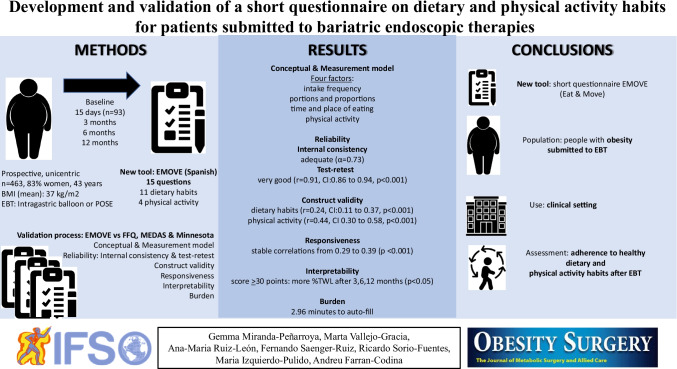

**Supplementary Information:**

The online version contains supplementary material available at 10.1007/s11695-021-05754-7.

## Background

The rapidly growing obesity epidemic [1] combined with the lack of effective dietary and pharmacological interventions and the risks of bariatric surgery [2–4] has led to an increased demand for endoscopic bariatric therapies (EBT) for weight loss, such as the intragastric balloon (IB) or the primary obesity surgery endoluminal (POSE) [5, 6]. Nevertheless, subjects with obesity submitted to EBT tend to regain weight over time [7] unless they adhere to healthy dietary and physical activity (PA) habits [8].

The evaluation of dietary and PA habits among individuals with obesity is essential to identify what modifications should be made to lose weight without relapse, especially if the person has undergone EBT to lose weight, which can be the last resort after many years of trying to lose weight with failed diets [9] and poor adherence to PA programs [10]. There are several questionnaires for evaluating dietary intake, such as a food frequency questionnaire (FFQ) developed by Martin-Moreno et al. [11] and the Mediterranean Diet Adherence Screener (MEDAS) developed in the PREDIMED Study, both validated for the Spanish population [12]. There are also questionnaires to assess energy expenditure associated with PA, such as the Minnesota Leisure Time Physical Activity Questionnaire (Minnesota), also validated for the Spanish population [13, 14]. While all are excellent and accurate questionnaires, they are time-consuming for participants, as is the FFQ, or require a trained interviewer, as is the case with the Minnesota and MEDAS questionnaires. Long questionnaires may also require the patient to complete them elsewhere, increasing the non-response rate [15].

In time-limited settings, such as the clinical practice, short questionnaires are needed for a rapid assessment of dietary and PA habits to evaluate the primary goal of a weight loss intervention [16]. Furthermore, subjects with obesity who have undergone EBT have a combination of conditions and particularities that should be considered, such as (i) a decreased stomach capacity leading to smaller food portions [5] and (ii) risk of fat-free mass loss due to a possible very low initial calorie intake that should be avoided through PA [17].

Therefore, the objective of this study was to develop and validate a short, self-administered scoring questionnaire to assess the level of adherence quickly and easily to healthy dietary and PA habits in subjects with obesity submitted to EBT. The ultimate goal is to provide a reliable and validated questionnaire that can be used routinely in clinical practice. The questionnaire would help to know the evolution of adherence to healthy habits (diet and physical activity) that the subject must acquire after the endoscopic intervention to achieve a healthy weight and not relapse into it. The questionnaire was called EMOVE, an acronym from **E**at and **Move**.

## Materials and Methods

### Participants

**Fig. 1:**
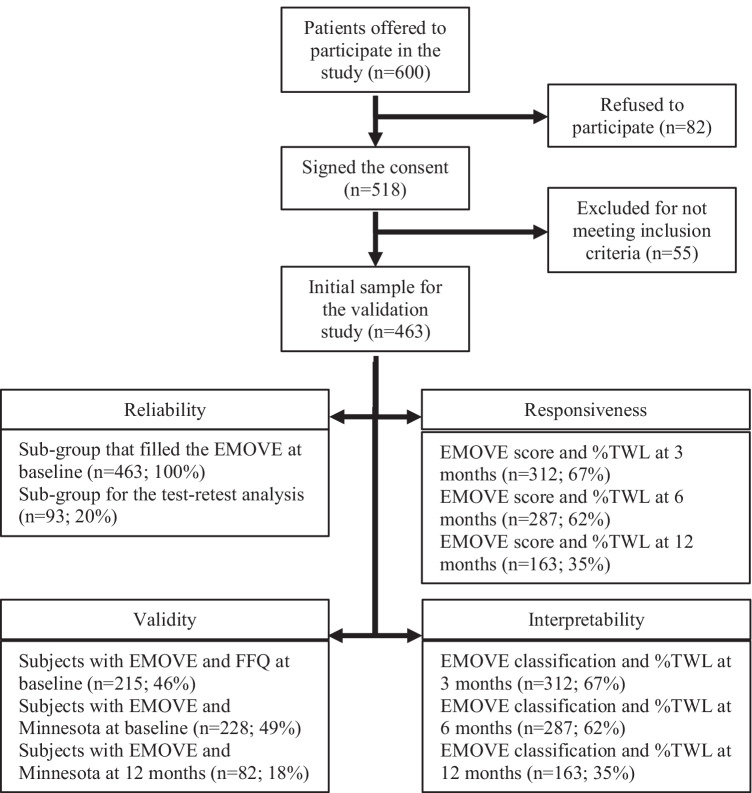
Flow diagram of the study recruitment process and completion rates (%) for the validation of the EMOVE questionnaire

The prospective, unicentric validation study was conducted in an adult population with obesity (18 to 64 years old) who attended a private clinic for a multidisciplinary treatment to lose weight with EBT (IB for 6 months or 12 months or POSE). Inclusion criteria were (i) speak Spanish, (ii) age between 18 and 64 years old, and (iii) not having been diagnosed with binge eating disorder or bulimia. Participation in the study was offered to 600 patients, of whom 82 refused to participate and 518 signed the informed consent. Subsequently, 55 patients were excluded because they did not meet the inclusion criteria. Finally, 463 patients were eligible and included in the study (Fig. 1).

### Collection of Data

Data collection began in January 2017 and lasted until August 2018, and the study included a 12-month follow-up. At the preoperative visit, height (m), using a fix wall stadiometer Seca 213 (Seca, CA, USA), and weight (kg), with a Tanita® BC-418 (Tanita, Amsterdam, The Netherlands), were measured in all participants. Both measures were taken with the patients wearing light clothes and without shoes. Body mass index (BMI) was calculated as weight (kg) divided by height (m^2^). Post-operative weight loss was also expressed as a percentage of total weight loss (%TWL) following the formula: [weight lost/initial weight] × 100. In this visit, the EMOVE questionnaire was also given to the patient, in paper format, to fill out and return to a registered dietitian (RD). In addition, the RD interviewed the patient and filled out the Minnesota questionnaire. Finally, a FFQ was given to the patient along with detailed instructions on how to fill it out.

During follow-up visits with the RD, weight was measured, and participants self-completed the EMOVE at 3, 6, and 12 months after EBT for validation analysis. In addition, the RD interviewed the participant and filled out Minnesota questionnaire at the 12-month follow-up visit. For the test–retest reliability study, the EMOVE was re-administered 15 days after the EMOVE at baseline, to a subgroup of 93 patients, selected at convenience. Given that this analysis allows to verify that the scores remain stable over time, a subgroup of patients is sufficient without the need to burden the entire sample. Figure 1 summarizes the process of recruiting subjects for the study and also shows the response and completion rates for the validation process.

### Development of EMOVE

The EMOVE questionnaire was developed following the criteria of the Scientific Advisory Committee (SAC) of the Medical Outcome Trust on the evaluation of the psychometric qualities of medical instruments [18]. The EMOVE was developed with the assistance of a panel of 4 experts (2 RDs, 1 psychologist, and 1 obesity medical doctor, all of them specialists in EBT). The panel proposed questions organized into two sections: dietary and PA habits. For dietary questions, key aspects of the eating process were considered, such as meal timing, portion sizes, liquid intake, and place of eating. These aspects have been consistently related to maintaining healthy body weight and adapted to the characteristics of patients after an EBT [19–26]. The latest Global Recommendations for adults on Physical Activity for Health from the World Health Organization [27] were chosen for questions about PA habits. Before proceeding to the validation process, EMOVE was pre-tested on a randomly selected sample of participants (*n* = 25) and some of the questions were rewritten for better understanding [28].

### Validation Process of EMOVE

The EMOVE questionnaire was validated following the criteria of the Scientific Advisory Committee (SAC) of the Medical Outcome Trust [18]. Therefore, its conceptual and measurement model was studied and the test reliability (internal consistency and repeatability or test–retest reliability), construct validity, responsiveness, interpretability, and burden were evaluated.

#### Conceptual and Measurement Model

Exploratory factor analysis (EFA) was applied to a total of 15 questions (11 dietaries and 4 on PA) using the principal component method. The rotated matrix was extracted with varimax orthogonal rotation. Values greater than 0.3 were considered moderate and those greater than 0.5 as large [29].

#### Reliability: Internal Consistency and Test–Retest

Internal consistency was analyzed using Cronbach’s *α* coefficient and the impact of each scale item was assessed by calculating the Cronbach’s *α* when the item was removed. The test–retest reliability related to the stability of the instrument over time was analyzed with Pearson’s correlation between the scores obtained in the first and second administration of the EMOVE. Values greater than 0.70 were considered adequate [30].

#### Construct Validity

The construct validity was evaluated by studying the correlation between EMOVE with other reference questionnaires using Pearson’s correlations. First, the EMOVE scoring system was developed as follows: each habit performed 0 days/week received 0 points; 1–3 days/week received 1 point; 4–6 days/week received 2 points; and 7 days/week received 3 points. Thus, the 11-question EMOVE dietary habits score (from 0 to 33 points) was correlated with the MEDAS score (0 to 14 points). The MEDAS score was extracted from the self-completed FFQ at baseline by the patient, as suggested by Schröder et al. [12]. A greater adherence to the Mediterranean diet could be reflected in better dietary habits; thus, a positive correlation between EMOVE and MEDAS can be expected. In addition, the 4-question EMOVE PA score (0 to12 points) was correlated with Minnesota questionnaire. A positive correlation between EMOVE PA score and Minnesota was also expected. Correlation coefficients greater than 0.20 were considered adequate [29].

#### Responsiveness

The ability of EMOVE to predict weight loss was estimated with Pearson’s correlation between the EMOVE score (0–45 points: 33 points from the dietary habits section and 12 points from the PA habits section) and the evolution of the %TWL patient. The results were expected to show a statistically significant correlation between the EMOVE score and the %TWL and this association should be stable over time.

#### Interpretability

The score resulting from the EMOVE can also be used as a categorical variable to categorize patients as more or less adhered to healthy dietary and PA habits. Following the recommendations of Triandis [31], a considerably higher value was assigned to healthy habits performed more frequently than less frequently. Therefore, three categories were established as follows: 0–29 points, which is the range of scores obtained if one or more of all 15 healthy habits included in EMOVE was performed less than 4 days a week (poor habits); 30–38 points, obtained when at least all 15 healthy habits were performed 4 days a week (good habits); and 39–45 points, obtained when at least 13 healthy habits were performed every day of the week (excellent habits). Consequently, %TWL mean values were calculated according to the EMOVE categorization (poor habits, good habits, and excellent habits).

#### Burden

Burden was measured by the average time to fill out the EMOVE in a sample of randomly chosen patients (*n* = 30) with a Hanhart Alpha Chronometer (Switzerland).

### Statistical Methods

Normality was confirmed in all variables by histograms, Q-Q plots, and Shapiro–Wilk’s test. Descriptive sociodemographic data of participants were presented as percentages for categorical data and mean and SD for continuous variables. Differences in patients’ sociodemographic data were calculated using the chi-square test for categorical variables and the Student *t*-test for continuous variables. The convenience of performing an exploratory factor analysis was assessed with the Kaiser–Meyer–Olkin (KMO) measure of the sampling adequacy and the Bartlett’s test. The extraction method was performed with principal components and varimax rotation. For the construct validity, associations, and *p* values between EMOVE, MEDAS and Minnesota were tested using Pearson’s correlation. The extreme values were excluded (± 2 SD; 5% of patients) for no plausible high or low energy expenditure (collected with Minnesota) or no plausible high or low energy intake (collected with FFQ) [32]. The responsiveness was estimated with Pearson’s correlation between EMOVE score and the %TWL, adjusting by age, gender, and type of intervention (6 months or 12 months of IB or POSE). Since the EMOVE categorization (poor, good, and excellent habits) was hypothesized as a predictor of %TWL, general linear models (GLMs) were applied followed by Tukey’s post hoc comparison between EMOVE categorization and the evolution of %TWL. All statistical analyses were conducted using STATA version 14 (StataCorp, College 257 Station, TX, USA). Statistical significance was established at *p* < 0.05.

## Results

### Participants

Characteristics of the study participants are summarized in Table 1. Non-statistically significant differences were observed in the characteristics of the patients selected for the test–retest study with the main study group.

**Table.1:** Characteristics of the participants in the study

	Participants completed EMOVE at baseline (*n* = 463)	Participants in the test–retest of EMOVE (*n* = 93)	*p* value
Age (years)	41.7 (10.1)	40.4 (9.8)	0.273
Sex (% women)	383 (82.7)	83 (89.2)	0.207
BMI (kg/m^2^)	37.1 (5.3)	37.0 (5.6)	0.279
Type of EBT (%)			
IB-6 months	133 (28.7)	28 (30.1)	
IB-12 months	203 (43.9)	39 (41.9)	0.235
POSE	127 (27.4)	26 (28.0)	
Education (%)			
No studies	7 (1.5)	0 (0.0)	0.104
Primary	118 (25.4)	15 (16.2)	
Secondary	174 (37.6)	35 (37.6)	
Technical	42 (9.1)	17 (18.3)	
University	112 (24.2)	20 (21.5)	
Unknown	10 (2.2)	6 (6.4)	

Mean (standard deviation, SD) for quantitative data and percentages for qualitative data. *p* values using χ^2^ for qualitative variables and Student *t*-test for quantitative variables. *BMI*, body mass index; *IB*, intragastric balloon; *POSE*, primary obesity surgery endoluminal.

### Development of EMOVE

The EMOVE developed by the panel of experts consists of 15 short, simple, and closed-ended questions (11 of dietary habits and 4 of PA habits) that measured the frequency of each evaluated event in a rating scale (never: 0 days/week; sometimes: 1–3 days/week; often: 4–6 days/week; always: 7 days/week) (Tables S1 and S2). Participants responded on their habits in the past month to minimize memory-related bias. The greater the final score, the better the patient’s adherence to the dietary and PA healthy habits.

### Validation Process of the EMOVE

#### Conceptual and Measurement Model

The Kaiser–Meyer–Olkin (KMO) measure of the sampling adequacy was found to be 0.71 while the Bartlett’s test concluded that the hypothesis of sphericity could be rejected (*p* < 0.001). These two values confirmed the convenience of performing an exploratory factor analysis (EFA), whose results are summarized in Table 2. Four significant components explaining more than 30% of the variation were extracted from the 15 questions of the EMOVE. The first component included questions related to PA (Q12, Q13, Q14, and Q15). The second included questions about the frequency of food intake (Q1, Q2, and Q3). The third component included questions related to the consumption of fruit and vegetables (Q5, Q6, Q7, and Q8). Finally, the fourth component contained questions of time and place of eating (Q4, Q9, and Q10). Liquid intake (Q11) was not included in any component.

**Table.2:** Results of the exploratory factor analysis (*n* = 463)

Item	Factor			
	1	2	3	4
Q12	0.78			
Q13	0.81			
Q14	0.52			
Q15	0.49			
Q1		0.33		
Q2		0.70		
Q3		0.73		
Q5			0.55	
Q6			0.37	
Q7			0.62	
Q8			0.37	
Q4				0.33
Q9				0.59
Q10				0.55

Correlations above ≥ 0.3 are shown.

#### Reliability: Internal Consistency and Test–Retest

Internal consistency evaluated with *α*-Cronbach, resulted in a value of 0.73 and partial results of 0.70 to 0.72 for each question (Table 3). The Pearson correlation coefficient between the EMOVE scores obtained in the first visit and after 15 days (test–retest) was 0.91 (95% CI: 0.86 to 0.94, *p* < 0.001).

**Table.3:** Impact of eliminating each scale item on Cronbach’s *α* (*n* = 463)

Item	*α* if question deleted
Question 1	0.72
Question 2	0.72
Question 3	0.71
Question 4	0.72
Question 5	0.70
Question 6	0.72
Question 7	0.71
Question 8	0.72
Question 9	0.71
Question 10	0.71
Question 11	0.72
Question 12	0.71
Question 13	0.71
Question 14	0.71

#### Construct Validity

The correlation between the EMOVE dietary score and the MEDAS was 0.24 (95% CI 0.11–0.37; *p* < 0.001; *n* = 215), while the coefficient between the EMOVE PA score and the Minnesota was 0.44 (95% CI 0.30–0.58; *p* < 0.001; *n* = 228). The same analysis was performed with data from the EMOVE and Minnesota at 12 months, obtaining a correlation of 0.50 (95% CI 0.38–0.62; *p* < 0.001; *n* = 82). In both cases, a sensitivity analysis was performed including the 5% of extreme cases from each side and resulted in non-significant differences with the result described above.

#### Responsiveness

Correlations between the EMOVE score and the evolution of the %TWL of participants over time were statistically significant (*p* < 0.001) as shown in Table 4. The correlation coefficients are significant at 3, 6, and 12 months, indicating that the association remains stable during the follow-up time. Although there is a slight increase in the magnitude of the coefficient, the differences between the three moments are not significant. In summary, all of this indicated that subjects with high EMOVE scores are more likely to have a consistently higher %TWL.

**Table.4:** Responsiveness of EMOVE to change of percentage of total weight loss (%TWL) in all participants after 3, 6, and 12 months after the EBT (IB and POSE)

Months of follow-up	*n*	Correlation coefficient (95% CI)	*p* value
3 months	312	0.29 (0.20, 0.39)	< 0.001
6 months	287	0.31 (0.21, 0.42)	< 0.001
12 months	163	0.39 (0.27, 0.50)	< 0.001

Adjusted by age, gender, and type of intervention (6 months or 12 months of IB and POSE)

#### Interpretability

As shown in Table 5, participants categorized as having good or excellent habits according to the EMOVE (score ≥ 30 points) lost significantly more weight (expressed as %TWL) than those who reported poor habits (score < 30 points) after 3, 6, and 12 months of the EBT.

**Table 5:** Mean (standard deviation) of the percentage of total weight lost (%TWL) according to EMOVE categorization after 3, 6, and 12 months after the EBT

	%TWL					
	3 months	*n*	6 months	*n*	12 months	*n*
Poor habits	9.3 (4.0)^a^	163	11.0 (5.9)^a^	149	11.1 (7.4)^a^	82
Good habits	10.7 (4.2)^b^	134	13.5 (5.5)^b^	119	15.9 (8.5)^b^	68
Excellent habits	13.4 (3.9)^c^	15	15.3 (6.02)^b^	19	20.1 (8.2)^b^	13

Poor habits: EMOVE score 0–29; good habits: EMOVE score 30–38; excellent habits: EMOVE score 39–45. Values with the same superscript in the same moment are not significantly different (*p* > 0.05) after the results of the Tukey’s post hoc test; adjusted by age, gender, and type of intervention (6 months or 12 months of IB or POSE)

#### Burden

The average time to complete the self-administered EMOVE was of 2.96 (± 0.52) min. Also, there were no missing answers since EMOVE was answered in the follow-up visit with the RD and any missed answer was filled.

Figure S3 summarizes the development and validation process of the EMOVE questionnaire, including the methodology applied (conceptual and measurement model, internal consistency and test–retest, validity, responsiveness, interpretability, and burden) and its results.

## Discussion

To our knowledge, the EMOVE represents the first validated short, self-administered scoring questionnaire in Spanish language to assess the adherence to healthy dietary and PA habits for patients submitted to EBT. The ultimate success of the EBT is the weight loss and its maintenance over time. For that, interventions that promote healthy dietary and PA habits are strongly recommended [33, 34]. Therefore, there is a need for simple, adequate, validated, easy-to-use, and easy-to-interpret tools, such as the EMOVE questionnaire, to evaluate the adherence to the intervention.

The EMOVE has been validated against two validated questionnaires: the FFQ, audited ad hoc with the MEDAS for the dietary habits, and the Minnesota questionnaire for the PA habits. The EMOVE has been created taking into account the need of validated short questionnaires rather than longer ones [35, 36]. Also, the concept of patient-reported outcomes, which nowadays is widely used to assess the success or failure of interventions in obesity [37], has been considered. In addition, by evaluating habits from the past month, EMOVE minimizes the limitations of measuring food intake and PA performance related to memory bias, as indicated in previous studies [38–41].

### Development of the EMOVE

Solid and emerging scientific evidence related to healthy dietary and PA habits have been used to develop the EMOVE items (Table S1) [42]. These were later confirmed by four significant components extracted by factor analysis. The first component included PA questions. There is evidence confirming adequate correlations between short questionnaires and accelerometers supporting the fact that a questionnaire could be useful for clinical settings [40, 43]. The second component included questions related to the frequency of food intake. A high frequency of snack consumption has been correlated with a lower diet quality [21, 44, 45] while having breakfast is related with a good quality diet and optimal energy balance, especially when stomach capacity is small [46, 47]. The third component included questions related to a high intake of vegetables and a low intake of animal products, which has been correlated with lower risk of adiposity and obesity [48, 49]. Finally, the fourth component grouped questions related to the time and place of eating which are associated with body mass index [21, 50]. The item related to water intake did not fit into any of the components; however, it was maintained in the EMOVE questionnaire as drinking water is widely recommended for both hydration and weight control [51, 52]. In addition, its inclusion did not affect the performance of the questionnaire. These four components were consistent with the topics of interest from the expert panel.

The EMOVE differs from other questionnaires to assess lifestyle issues previously developed and validated in Spanish population with obesity. For instance, Pardo et al. [53] in 2004 developed and validated a lifestyle questionnaire for Spanish people with overweight and obesity. However, the questionnaire contains outdated questions to be used now, such as the preference for low-fat dairy over whole dairy products, which are now known to have no correlation with obesity [54]. In addition, the questionnaire of Pardo et al. [53] is quite permissive with the intake of low-graduation alcohol beverages, like beer and wine, which are associated with overweight and obesity [55]. On the other hand, Castro Rodríguez et al. developed and validated a questionnaire appropriate for clinical practice based on Pardo’s questionnaire; however, none of them is specific for patients after EBT [56]. Recently, a self-filling questionnaire which allows to quantify the adaption to dietary/lifestyle suggestions provided after bariatric surgery has been validate for Italian population. Nevertheless, this new instrument does not include EBT patients, whose casuistic and evolution differ greatly from patients undergoing bariatric surgery [57].

### Validation of the EMOVE

The EMOVE showed adequate internal consistency and very good test–retest [30]. The time interval between evaluations was set at 2 weeks, which is often considered appropriate [58]. The subgroup of 93 patients for the test–retest, which represented a 20% of the study participants, was considered adequate [53, 59–62]. Furthermore, the Medical Outcome Trust guidelines do not establish a criteria formula for the desired sample size. The EMOVE also showed a moderate construct validity both for the section related to dietary habits and for the section related to PA. That was in accordance with previous publications [35, 42, 63]. The higher correlation found at 12 months in EMOVE PA part could be explained by the fact that people who participate in behavior change intervention are more aware of their routine behavior and, therefore, report it with greater precision.

It should be noted that the EMOVE questionnaire can predict the evolution of the percentage of TWL since there is a stable significant positive association between the EMOVE score and weight loss and, additionally, patients who had higher EMOVE scores (> 30 points), which correspond from good to excellent habits, lost significantly more weight after 3, 6, and 12 months of EBT. The interpretability of the EMOVE is consistent with previous evidence showing weight plateaus approximately 6 months after weight-loss interventions [64, 65]. Finally, the EMOVE showed an excellent administrative burden due to the short time invested by the participant (in average 2.96 min). Therefore, the EMOVE questionnaire can be useful in clinical settings, where longer questionnaires are not feasible and more sophisticated instruments are not affordable.

This study shows several limitations. First, the dependence on the participants’ memory to complete the FFQ at baseline, since they must remember the frequency and the amount of food and drinks ingested the previous year. The information may have also been influenced by the appropriateness of a given answer. Another limitation is that not all patients undergoing EBT accepted to complete the follow-up, which may increase bias since those patients who did not agree to participate in the study could potentially be the ones with the worst results after EBT. Nevertheless, the study has important strengths. First, the EMOVE has been developed in strict accordance with the Scientific Advisory Committee of the Medical Outcome Trust [18] on how to assess the psychometric qualities of a medical instrument and covered most of the recommended aspects. Second, it has been validated against a relatively large sample of subjects with obesity undergoing EBT. In addition, by having long-term patient data, we were able to assess the sensitivity of the EMOVE to predict weight change.

## Conclusions

We have developed and validated a short, self-administered scoring 15-item questionnaire that allows to quantify the patient’s adherence to the indications of healthy dietary and PA habits provided after EBT. The EMOVE is quick and easy for the patient to respond to, as well as to be interpreted by the multidisciplinary team responsible for the behavior change intervention. Important questions around how, rather than what, patients eat, and move are addressed. The EMOVE could be useful to optimize the time invested in each patient in the follow-up visits. Based on our clinical experience, the low burden of administration, less than 3 min, helps make the follow-up visit more cost-effective, as visits are generally time-limited and patients often must invest time and money to attend (transportation, waiting room, and the visit itself).

## Supplementary Information

Below is the link to the electronic supplementary material.

Supplementary file1 (DOCX 41.4 KB)
