# Supplementary material for: Development and Validation of a Short Questionnaire on Dietary and Physical Activity Habits for Patients Submitted to Bariatric Endoscopic Therapies
Source: Obes Surg. 2021 Oct 19;32(1):142–51. doi: 10.1007/s11695-021-05754-7 (PMC8752550; doi:10.1007/s11695-021-05754-7)
Supplement: Supplementary file 1 — Supplementary file1 (DOCX 41.4 KB) [file 11695_2021_5754_MOESM1_ESM.docx]

**EMOVE. EAT & MOVE CUESTIONARIO CORTO DE HÁBITOS SALUDABLES DE ALIMENTACIÓN Y ACTIVIDAD FÍSICA EN PACIENTES CON OBESIDAD SOMETIDOS A UNA INTERVENCIÓN ENDOSCÓPICA**

**Nombre y apellidos: ________________________________________________ Fecha_______________**

**IMPORTANTE**: considere **NUNCA**: ningún día de la semana; **A VECES**: 1-3 días a la semana; **GENERALMENTE**: 4-6 días a la semana; **SIEMPRE**: 7 días a la semana. **VALORE EL MES ANTERIOR**

ALIMENTACIÓN

1. **¿Desayuna antes de salir de casa?** (considere desayunar alguna cosa consistente como lácteo, cereal y/o fruta)

Nunca A veces Generalmente Siempre

1. **¿Merienda a media tarde?** (considere merendar alguna cosa consistente como lácteo, cereal y/o fruta)

Nunca A veces Generalmente Siempre

1. **¿Come entre 4 y 5 veces al día?** (considere 1 día 24h)

Nunca A veces Generalmente Siempre

1. **¿Las comidas las hace en horas similares?** (por ejemplo, come cada día a las 14h)

Nunca A veces Generalmente Siempre

1. **¿Come un mínimo de 1 ración (80g) de verdura cocida o cruda en cada comida y cena?**

Nunca A veces Generalmente Siempre

1. **En caso de hacer dos platos,** **¿el segundo plato es más reducido que el primer plato?**

Nunca A veces Generalmente Siempre

1. **En caso de hacer plato único,** **¿las verduras y hortalizas ocupan la mitad del plato?**

Nunca A veces Generalmente Siempre

1. **En caso de hacer postres,** **¿escoge postres a base de fruta fresca?**

Nunca A veces Generalmente Siempre

1. **¿Come principalmente alimentos caseros?** (por ejemplo, evita los enlatados, evita la comida preparada, evita las pizzas comerciales, evita pedir comida china para llevar)

Nunca A veces Generalmente Siempre

1. **¿Come y/o cena principalmente en casa o con *tupper*?** (por ejemplo, evita los comedores laborales, evita los bares y restaurantes, evita el *fastfood*)

Nunca A veces Generalmente Siempre

1. **¿Bebe principalmente agua?** (por ejemplo, evita o modera la cola, tónica, agua con sabores, zumos de fruta, cerveza, vino, ginebra)

Nunca A veces Generalmente Siempre

**IMPORTANTE**: Valore los momentos de ocio, de desplazamiento, de trabajo, de tareas del hogar, de deporte o entrenamiento programado, de juegos. Considere **ACTIVIDAD AERÓBICA MODERADA**: aquella que mientras la realiza le permite hablar con normalidad; **ACTIVIDAD AERÓBICA VIGOROSA**: mientras la realiza habla con dificultad. **VALORE EL MES ANTERIOR**

ACTIVIDAD FÍSICA

1. **¿Acumula un mínimo de 150 minutos semanales (2:30h) de actividad aeróbica moderada o 75 minutos semanales (1:15h) de actividad aeróbica vigorosa?** (por ejemplo, caminar a paso vivo, correr, montar en bicicleta, nadar)

Nunca A veces Generalmente Siempre

1. **¿Acumula un mínimo de 300 minutos semanales (5h) de actividad aeróbica moderada o 150 minutos semanales (2:30h) de actividad aeróbica vigorosa?** (por ejemplo, caminar a paso vivo, correr, montar en bicicleta, nadar)

Nunca A veces Generalmente Siempre

1. **¿Realiza actividad aeróbica, moderada o vigorosa, durante al menos 10 minutos seguidos?** (por ejemplo, caminar a paso vivo, correr, montar en bicicleta, nadar)

Nunca A veces Generalmente Siempre

1. **¿Realiza actividades de fortalecimiento muscular 2 o más veces por semana?** (por ejemplo, subir escaleras, coger pesos, entrenamiento)

Nunca A veces Generalmente Siempre

**Nunca: 0 puntos; A veces: 1 punto; Generalmente: 2 puntos; Siempre: 3 puntos. Buenos hábitos 30 puntos o más**

**EMOVE. EAT & MOVE SHORT QUESTIONNAIRE ON DIETARY AND PHYSICAL ACTIVITY HEALTHY HABITS IN PATIENTS WITH OBESITY SUBMITTED TO BARIATRIC ENDOSCOPIC THERAPIES**

**Name and last name: ________________________________________________ Date: _______________**

**IMPORTANT**: **NEVER**: 0 days per week; **SOMETIMES**: 1-3 days per week; **USUALLY**: 4-6 days per week; **ALWAYS**: 7 days per week. **CONSIDER LAST MONTH’S HABITS**

DIETARY

1. **Do you have breakfast before leaving home?** (Consider breakfast something consistent like dairy, cereal and/or fruit)

Never Sometimes Usually Always

1. **Do you have an afternoon snack?** (Consider snack something consistent like dairy, cereal and/or fruit)

Never Sometimes Usually Always

1. **Do you eat 4-5 times a day?** (Consider a 24h day)

Never Sometimes Usually Always

1. **Do you eat meals at similar times?** (For example, have lunch every day at 2:00 p.m.)

Never Sometimes Usually Always

1. **Do you eat at least 1 serving (80 g) of cooked or raw vegetables with every meal?**

Never Sometimes Usually Always

1. **If you eat (or have) two courses, is the second course smaller than the first course?**

Never Sometimes Usually Always

1. **If you eat just a plate, do vegetables (raw or cooked) take up half of the plate?**

Never Sometimes Usually Always

1. **If you eat dessert, do you prefer fresh fruit-based dessert?**

Never Sometimes Usually Always

1. **Do you eat mostly homemade foods?** (For example: avoid canned food, ultraprocessed- food, commercial pizzas, ordering Chinese food takeout)

Never Sometimes Usually Always

1. **Do you have lunch and/or dinner mainly at home or in a food-container?** (For example: avoid canteens, bars, restaurants, fast-food restaurants?

Never Sometimes Usually Always

1. **Do you drink mainly water?** (For example: avoid or moderate the consumption of soft drinks, flavored waters, fruit juices, beer, wine or gin)

Never Sometimes Usually Always

**IMPORTANT**: Value moments of leisure, travel, work, housework, sports or scheduled training, games. Consider **MODERATE AEROBIC ACTIVITY**: while doing it allows you to speak normally; **VIGOROUS AEROBIC ACTIVITY**: while doing it makes it difficult to speak. **CONSIDER LAST MONTH’S HABITS**

PHYSICAL ACTIVITY

1. **Do you build up to at least 150 minutes throughout the week (2:30h) of moderate-intensity aerobic physical activity or do at least 75 minutes throughout the week (1:15h) of vigorous-intensity aerobic physical activity?** (For example, walking at a good pace, running, biking, swimming)

Never Sometimes Usually Always

1. **Do you build up to at least 300 minutes throughout the week (5h) of moderate-intensity aerobic physical activity or do at least 150 minutes throughout the week (2:30h) of vigorous-intensity aerobic physical activity?** (For example, walking at a good pace, running, biking, swimming)

Never Sometimes Usually Always

1. **Do you perform this moderate-intensity or vigorous-intensity aerobic physical activity in bouts of at least 10 minutes duration?** (For example, walking at a good pace, running, biking, swimming)

Never Sometimes Usually Always

1. **Do you engage in muscle-strengthening activities 2 or more days a week?** (For example, climbing stairs, carrying weight, training sessions)

Never Sometimes Usually Always

**Never: 0 points; Sometimes: 1 point; Usually: 2 points; Always: 3 points. Good habits 30 points or more**

Figure S3. Development and validation process with the methodology applied and its results.
